# Lectures on the Diseases of Infancy and Childhood

**Published:** 1860-05

**Authors:** 


ART. VII.—*Lectures on the Diseases of Infancy and Childhood.* By CHARLES WEST, M.D. Third American edition. pp. 629. Philadelphia: Blanchard & Lea, 1860.

DR. WEST has long been favorably known in this country as the author of an excellent treatise on the "Diseases of Women," and also by the present publication, originally issued at London, in 1848. "I have done my best," says the author, in his preface, "in this edition, which is much enlarged, to supply past deficiencies, as well as to correct former errors;" and it is proper to add that the value of the work has been materially augmented by appending some lectures published by Dr. West since the appearance of the fourth London edition, of which the American is a reprint. As it now presents itself, it is unquestionably the most original, if not also the most complete treatise on the diseases of children, in the English language, founded as it is upon the results of 900 observations, and 283 dissections made among nearly 30,000 infants examined by the author during the last twenty years. The style of the work is at once elegant, clear, and forcible, and a careful perusal of its opulent pages cannot fail to satisfy every one that it is the production of one of the master-spirits of the profession.

Anything even like an attempt at an analysis of this able work would, at this late period, be obviously out of place. Nevertheless, we cannot refrain from extracting the following passage on puncture of the cranium as a means of cure in chronic hydrocephalus, inasmuch as the sentiments expressed in it so fully accord with the results of our own observations. The passage contains no allusion to the operations of Dr. Conquest, who published, many years ago, a statement to the effect that it had proved successful in his hands not less than eighteen times. "*Credat Judæus Apella, non ego.*"

"Puncture of the cranium, and the evacuation of the fluid, is another proceeding which has been occasionally resorted to from a very early period in the history of medicine, and which is even at the present day strongly advocated by some writers; not merely as a palliative measure, or as an adjunct to other remedies, but as a means of effecting the radical cure of the disease. Opinion, however, is much divided as to the propriety of this practice, the statistics of which certainly do not yield any very encouraging results. Fifty-six cases, the particulars of which I published some years ago,* as I found them recorded in various publications, yielded a proportion of fifteen alleged recoveries; but on subjecting these cases to a rigid analysis, it appeared that in only four of this number were the particulars recorded with sufficient accuracy, or had the interval since the performance of the operation been long enough to warrant our admitting them as permanent cures. The very unfavorable conclusions which I then expressed with reference to this operation were afterwards criticised by M. Durand-Fardel,† a gentleman whose opinion on any question connected with cerebral disease is entitled to very great weight. He observed that, while it is admitted that in a few cases puncture of the cranium has been followed by complete and permanent cure, its failure on other occasions was often manifestly due to the existence of utterly incurable malformation of the brain; while in many instances, though the operation failed to effect a cure, yet the very frequency with which it was repeated proved that in itself it is not usually attended with any considerable danger. Since, then, it may do good—since, if it should fail, its failure is often due to causes which no remedy could remove—since, even if it should do no good, yet in the majority of instances it will do no harm, while if left to itself the course of the disease is almost invariably to a fatal result, he advocates its performance in cases of chronic hydrocephalus. Though I cannot but fear that this gentleman rather underrates the amount of immediate risk attendant on the operation, yet I think that his authority ought at least to have so much weight with you as to prevent your looking upon its performance as altogether unjustifiable, and the rather since there is good reason for believing that the accumulation of fluid in the ventricles is frequently the result of previous inflammation of their lining membrane, and that puncture of the cranium may therefore contribute to the cure of dropsy of the brain, just as tapping the abdomen does to the cure of ascites."

* In the Medical Gazette, April, 1842.

† In the Bulletin Générale de Thérapeutique, vol. xxiii. p. 190.

